# Supplementary material for: Structural modularity of the XIST ribonucleoprotein complex
Source: Nat Commun. 2020 Dec 2;11:6163. doi: 10.1038/s41467-020-20040-3 (PMC7710737; doi:10.1038/s41467-020-20040-3)
Supplement: Supplementary file 1 — Supplementary Information [file 41467_2020_20040_MOESM1_ESM.pdf]

Supplementary Information

Structural modularity of the XIST ribonucleoprotein complex

Lu *et al.* 2020.

This file contains the following information: Supplementary Figures 1-6, Supplementary Methods (as supplementary Notes 1-6) and Supplementary Discussion.

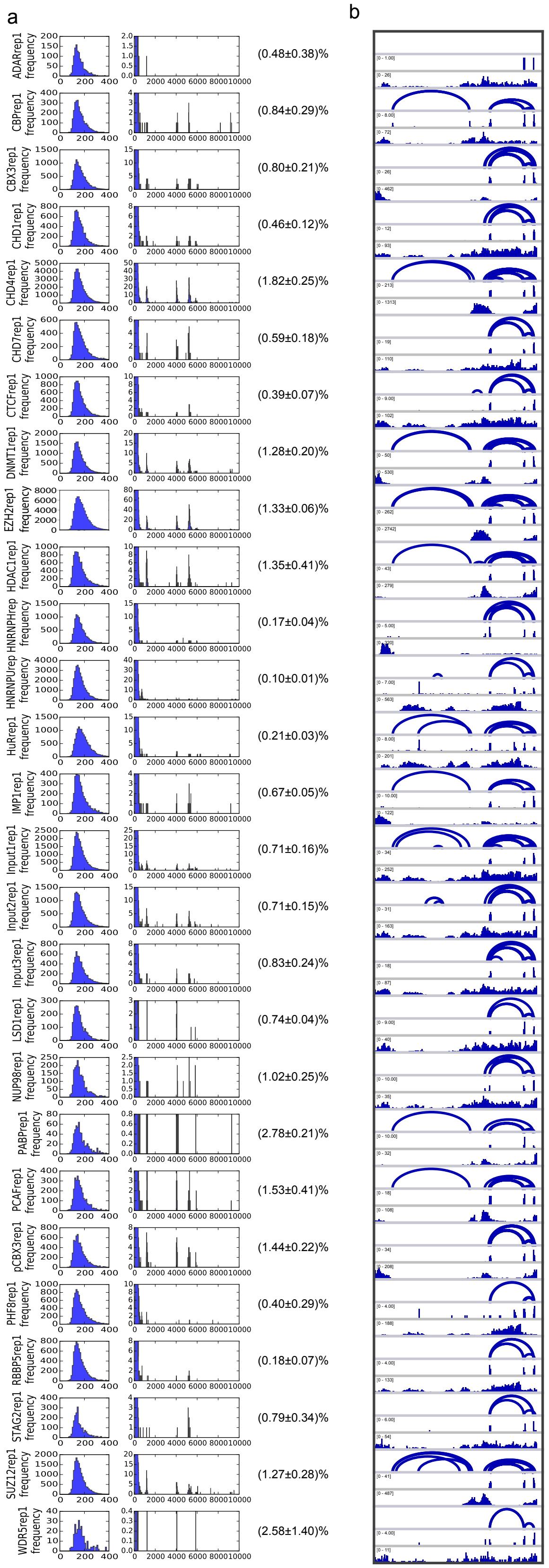

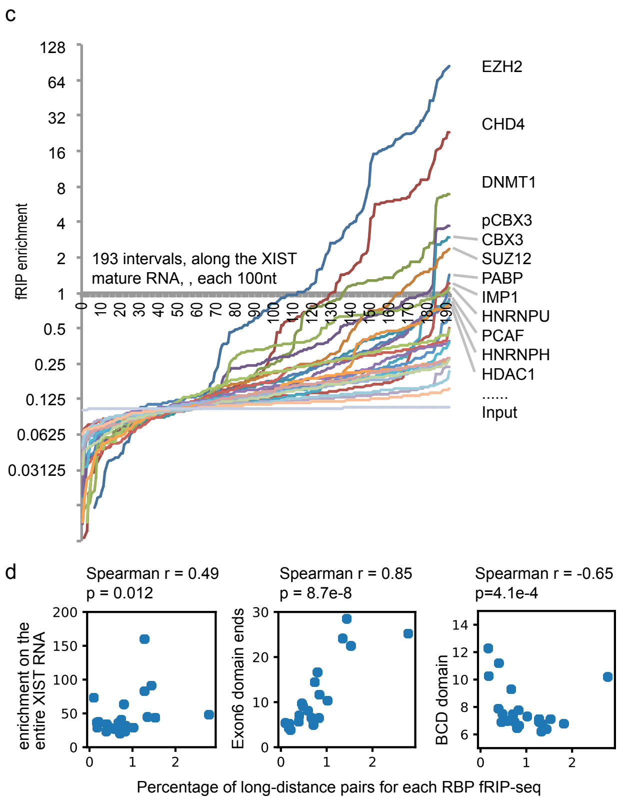

Supplementary Figure 1. fRIP-seq data for all proteins. Related to Figure 1. a, Distance distribution of the short-distance and long-distance read pairs from paired-end fRIP-seq data. See Figure 1 for description of the panels. Here one replicate is selected from each control or fRIP-seq experiment. The percentage of long distance read pairs in all read pairs, calculated as average ± standard deviation are shown to the right of the panel (n=2 for EZH2 and STAG2, n=3 for the others). **b,** IGV genome browser tracks for all the fRIP-seq data, showing one replicate from each experiment. For each sample, the ¬1st track, geometric_long_arc.bed, presents the connections of the two sequencing tags in each paired-end read; the second track, geometric_long.bedgraph, presents the depth of long-distance read pairs (>1000nt); the third track, geometric.bedgraph, presents the depth of all reads mapped to XIST. **c,** Enrichment of proteins on XIST in 100nt bins, sorted in ascending order in log scale. The top enriched proteins and input are labeled. The X-axis indicates the ranks of the 193 bins. **d,** Correlation of the percentage of long-distance pairs with enrichment of each RBP on the entire XIST RNA (left panel), or the ends of Exon6 domain (ranges 11200-14000 and 17500-19300, middle panel), or the middle of BCD domain (range 3000-10000, right panel). The strongest positive correlation with the Exon6 domain ends makes sense because the long-distance pairs are primarily connecting the Exon6 domain ends. Source data are provided as a Source Data file.

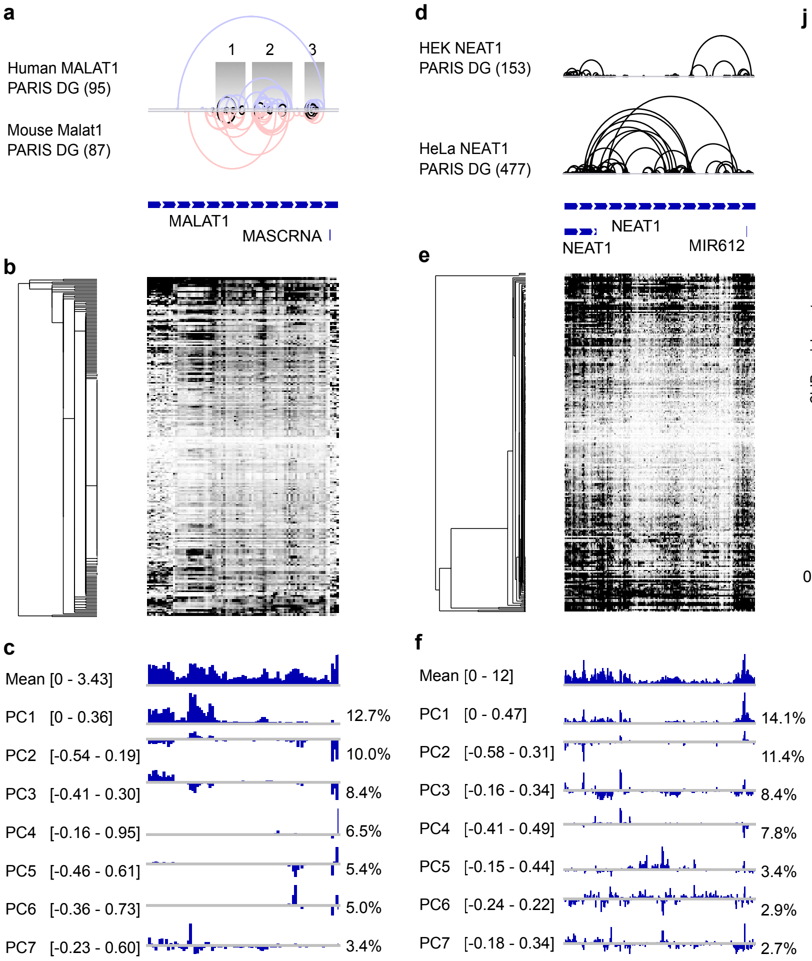

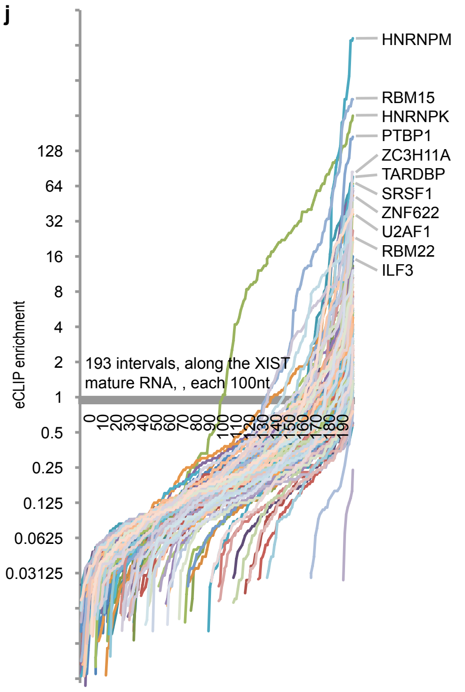

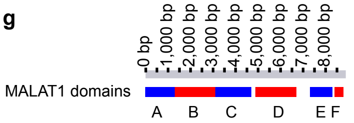

h Protein enrichment on MALAT1 (eCLIP)

A: 19	ratio
HNRNPK	94.57
SLTM	31.09
SAFB2	21.93
YWHAG	20.05
TIA1	19.41
HNRNPU	16.98
PPIL4	13.97
AKAP8L	6.97
FUS	6.95
HNRNPA1	6.7
DGCR8	6.57
SAFB	5.81
MTPAP	5.6
HNRNPM	5.37
DDX21	3.43
DDX55	3.33
LIN28B	3.14
DROSHA	3.12
HNRNPUL1	2.67

B: 29	ratio
ZNF800	235.11
TRA2A	142
HLTF	36.51
SDAD1	33.51
RBM15	26.76
PUM1	14.76
BUD13	13.4
GPKOW	11.72
NIPBL	11.28
AGGF1	10.24
ZNF622	8.94
SSB	8.22
RPS3	7.73
EIF4G2	7.6
UCHL5	7.35
GRWD1	7.28
LARP4	6.55
AQR	5.8
DDX24	5.54
KHDRBS1	5.45
XRCC6	5.31
WDR43	4.19
UTP18	4.14
NSUN2	4.12
AKAP1	3.66
SRSF7	3.49
FASTKD2	3.26
UTP3	3.21
ABCF1	2.94

C: 11	ratio
APOBEC3C	19.75
EIF3G	7.08
SF3B1	5.45
FAM120A	4.44
PRPF8	4.42
FXR1	4.36
FTO	3.68
TROVE2	2.95
NPM1	2.92
DDX6	2.72
EXOSC5	2.52

D: 8	ratio
TARDBP	49.82
GNL3	16.05
MATR3	6.08
HNRNPL	6
DHX30	3.96
IGF2BP1	2.84
METAP2	2.71
UPF1	2.57

E: 8	ratio
YBX3	42.9
PUS1	9.39
QKI	7.49
PHF6	6.25
IGF2BP2	4.43
FXR2	3.92
LARP7	3.1
SBDS	2.62

F: 39	ratio
SF3B4	224.5
SMNDC1	133.38
PABPC4	83.09
U2AF2	71.13
KHSRP	67.47
ZC3H11A	52.61
WRN	27.07
PTBP1	26.34
SUPV3L1	24.94
CPSF6	20.54
DDX51	15.63
ZRANB2	14.57
DDX42	14.47
WDR3	13.91
EFTUD2	13.88
FMR1	13.29
LSM11	12.81
CSTF2T	11.57
NOLC1	11.55
ZC3H8	9.76
RBFOX2	9.49
U2AF1	8.6
GTF2F1	8.32
AARS	6.96
RBM22	6.94
CPEB4	6.71
RPS11	5.21
XRN2	5.19
NONO	4.37
RBM27	3.98
DDX3X	3.69
EWSR1	3.33
ILF3	3.29
SRSF1	3.2
PCBP1	3.1
DDX52	2.98
TBRG4	2.87
GEMIN5	2.82
PUM2	2.64

i fRIP-seq

A: 3	ratio
HNRNPU	6.78
CTCF	2.91
CHD1	2.28

B: 3	ratio
HDAC1	9.66
CHD4	2.89
CBX3	2.29

C: 1	ratio
HNRNPH	4.21

D: 2	ratio
SUZ12	2.47
RBBP5	2.94

E: 1	ratio
HuR	2.88

F: 8	ratio
PABP	15.71
WDR5	3.24
EZH2	8.34
CHD7	7.22
CBP	8.09
PCAF	3.21
DNMT1	3.84
Input3	2.08
PHF8	12.56

Supplementary Figure 2. eCLIP analysis of protein binding on MALAT1 and NEAT1. a,d, PARIS derived structure models and gene models for MALAT1 (a) and NEAT1 (d) from 27. **b,e**, Clustering of protein enrichment profiles in 100nt windows for all 242 samples of the 121 proteins, two biological replicates each, for MALAT1 (b) and NEAT1 (e). **c,f,** PCA analysis of all eCLIP data in 100nt windows for MALAT1 (c) and NEAT1 (f). The mean and first 7 principal components are displayed together with percentage of variation explained by each component on the right. Together, these principal components explain 51% of total variation in each lncRNA. **g**, RNP domains in MALAT1 based on PARIS and fRIPseq/eCLIP clustering analysis. The coordinates are as follows. A: 0-1300, B: 1300-3100, C: 3100-4700, D: 4900-6700, E: 7300-8300, F: 8400-8800. **h-i**, Enrichment ratios of proteins on the 6 domains (A-F) in MALAT1 based on eCLIP (panel h) or fRIP-seq (i). For each protein, the enrichment on 6 domains are normalized so that the lowest is 1 and the highest one, if larger than 2, is designated as the specific domain that bind the protein. Most proteins are associated with domains A, B and F. Domains C-E, which corresponds to the previously defined domains 1-3 based on PARIS, have less associated proteins. **j**, Enrichment of proteins on XIST in 100nt bins based on eCLIP, sorted in ascending order in log scale. The top enriched proteins and input are labeled. The X-axis indicates ranks of the 193 bins.

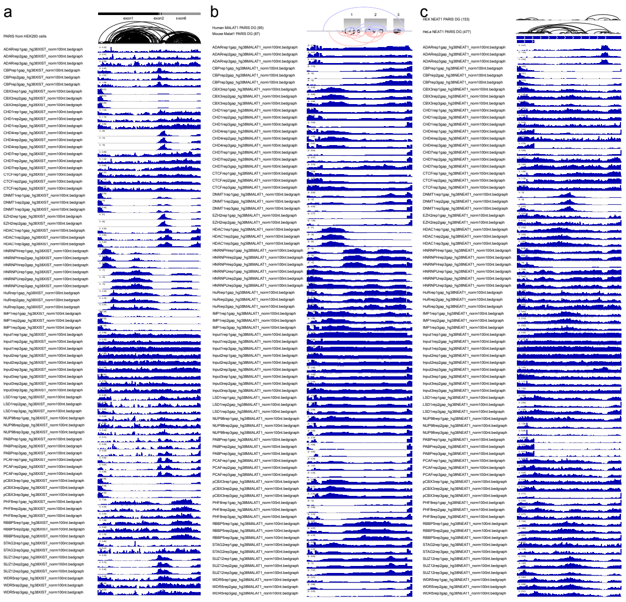

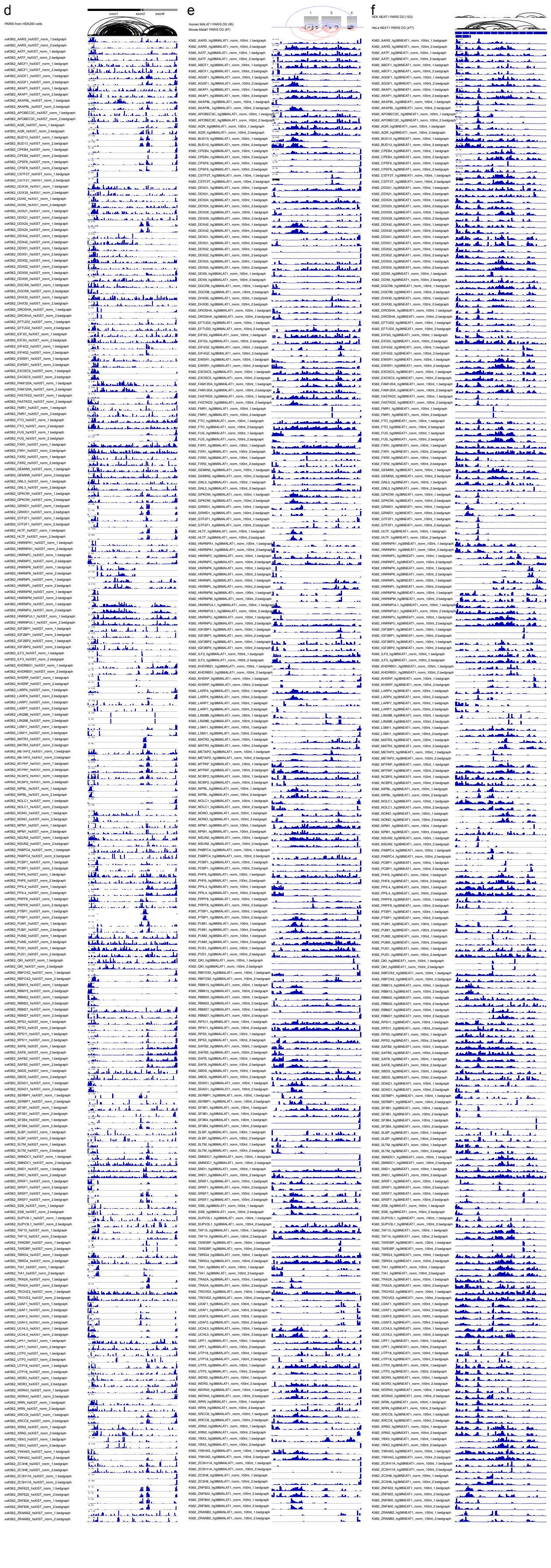

Supplementary Figure 3. fRIP-seq and eCLIP enrichment tracks for XIST, MALAT1 and NEAT1. a-c, Enrichment tracks for 79 fRIP-seq samples (duplicates or triplicates for each protein) on XIST, MALAT1 and NEAT1, in 100nt bins. Each track was normalized against background. See Methods for details. **d-f**, Enrichment tracks for 242 eCLIP samples (duplicates for each protein) on XIST, MALAT1 and NEAT1, in 100nt bins. Source data are provided as a Source Data file.

Modular architecture of lncRNAs MALAT1 and NEAT1. In order to determine whether the modular architecture is a general principle in the organization of large RNP complexes, we applied clustering and PCA analysis to MALAT1 and NEAT1, two lncRNAs that are highly expressed and implicated in nuclear body formation and splicing regulation. We observed clear RBP associated RNA domains in MALAT1, but the domain definitions for NEAT1 are not as clear as XIST and MALAT1 (Supplementary Figure 2a-i). For example in MALAT1, we observed 6 major RBP-defined domains, where 3 of them associate with the PARIS-derived RNA domains (Supplementary Figure 2g-i, see Supplementary Figure 3 for all fRIP-seq and eCLIP tracks). Interestingly, the first two RBP-derived domains are not associated with strong RNA structures. This difference among the lncRNAs XIST, MALAT1 and NEAT1 suggests that the structural organizations of lncRNAs are diverse.

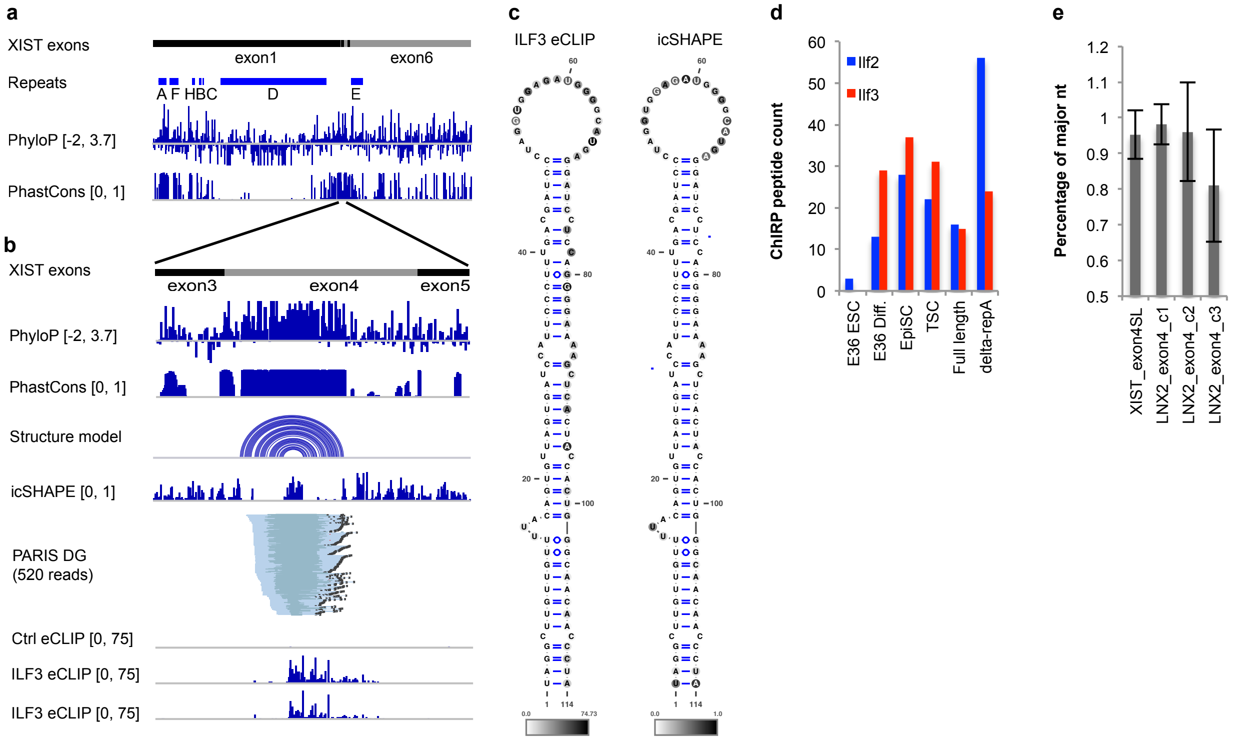

Supplementary Figure 4. Structure and interaction model of the conserved Exon 4 stemloop. a, Annotation of the human XIST mature transcript. The 6 exons in human XIST were concatenated, with the introns removed. The repeat elements were annotated based on 46. The placental mammals phyloP and 100 vertebrates PhastCons were from UCSC. **b,** XIST exon 4 forms a conserved stemloop structure that interacts with ILF3. The icSHAPE and PARIS data were from human HEK293T cells 27, while the ILF3 eCLIP data were from human K562 cells 36. ILF3 binds the loop and right side of the stem. Note, the stem-loop region in exon4 is highly conserved, while the rest of exon4 is not. **c,** The icSHAPE and ILF3 eCLIP data are plotted on the human XIST exon 4 stemloop structure. **d,** Both ILF3 and ILF2 were previously identified as XIST interactors 22. Peptide counts from XIST ChIRP-MS were plotted against cell lines used that indicate different stages in stem cell differentiation. One mass-spec dataset was available for this analysis. **e,** The conservation of the stemloop region in exon4 in eutherian mammals as compared to the ancestral LNX2 exon4 region. Percentage of the dominating nucleotide at each position was calculated. LNX2 third codon position is less conserved consistent with the wobble position. Data are presented as mean values +/- standard deviation in the bar graph. n=116 nucleotide positions for XIST_exon4SL. n=67 for the rest. p-value=5.2E-10 for the two-sided t-test between XIST_exon4SL and LNX2_exon4_c3. Source data are provided as a Source Data file.

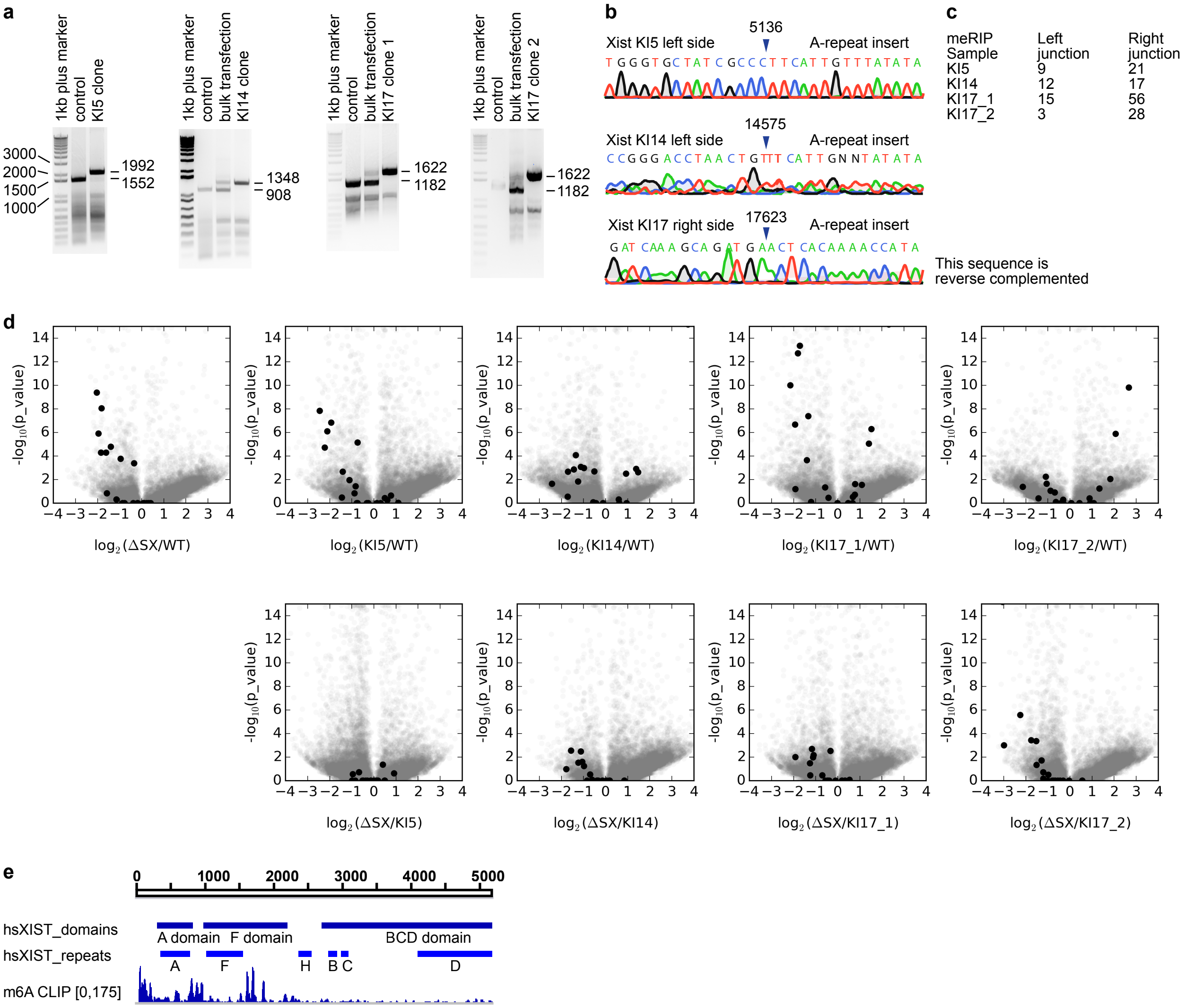

Supplementary Figure 5. Relocation of the A domain alters m6A modification patterns. Related to Figure 4. a, Genotyping PCR for the four clones picked for A-repeat relocation. Control samples are the starting ΔSX cell line (A repeat deletion). PCR for bulk transfections were performed after CRISPR editing and before picking clones. Molecular size markers are in base pairs. These results were not repeated since the bands are clearly indicating successful editing. In addition, the results were confirmed in panels (b) and (c), using Sanger sequencing and high throughput sequencing. **b,** Sanger sequencing confirming the insertion of the A-repeat region in the correct orientation to the 3 new locations. Each side of the junction shows 15nt. The traces from the sequencing report pdf were joined (the end of line after TGCTA for KI5, CTGTTT for KI14 and AGCAGA for KI17). **c,** Junction reads cannot be mapped to the wild type Xist, and are instead extracted from the raw fastq data as follows. KI5: GCTATCGCCCTTCATTGTTT (KI5_left+Arepeat_left) and TTTTGTGAGTCAGGTCACAT (Arepeat_right+KI5_right). KI14: ACCTAACTGTTTCATTGTTT (KI14_left+Arepeat_left) and TTTTGTGAGTTG-GCTTTATC (Arepeat_right+KI14_right). KI17: CATCCTACCATTCATTGTTT (KI17_left+Arepeat_left) and TTTTGTGAGTTCATCTGCTT (Arepeat_right+KI17_right). Despite the low coverage in the insertion sites, we obtained between 3-56 reads spanning each of the 6 junctions for the 3 insertion sites. **d,** Global changes of m6A modifications after relocation of the A-repeat domain. m6A modification sites were identified using the m6aViewer software (version 1.6.1) with the default parameters and negative strand bam files (half the data). Differences were visualized in volcano plots in the selected pairs of comparisons. Log scale fold changes (LFC) and negative log scale p values were used as the x-axis and y-axis, respectively. The black dots represent the m6A modification sites in Xist, as automatically detected by m6aViewer. The A-repeat deletion results in loss of most m6A modification sites (ΔSX/WT), whereas relocation of A-repeat results in partial rescue of some m6A sites (first row, KI5/WT, KI14/WT, KI17_1/WT and KI17_2/WT, see the black dots on the right side of the volcano plots). Compared to the A-repeat deletion line (ΔSX), the relocation of A-repeat in most cases results in higher m6A levels (second row, on the left side of each volcano plot, since the values are plotted as ΔSX/KI). The m6A levels are more scattered than the results shown in Figure 4. This is because m6aViewer reports m6A sites based on an automatic detection, where several closely spaced sites correspond to one m6A domain based on our manual annotation. The aggregation of m6A sites in the manually defined m6A domains leads to bigger accumulated difference higher statistical significance. One replicate was available for each sequencing library. **e,** Zoom-in view of the m6A CLIP data from Fig. 4a. The A-repeat domain itself is not methylated, rather it is the surrounding sequences that are. Source data are provided as a Source Data file.

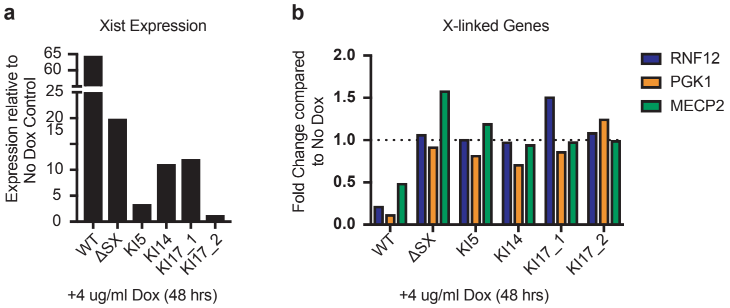

Supplementary Figure 6. Analysis of X chromosome inactivation after relocation of the A-repeat. Related to Figure 4. a, A-repeat relocation cell lines were induced to express Xist with doxycycline treatment and Xist levels were measured using qRT-PCR. **b,** A-repeat relocation cell lines were induced to express Xist. Then the expression of X-linked genes were measured using qRT-PCR. Source data are provided as a Source Data file. One replicate was quantified for each condition.

Supplementary References:

1. Frieda, K.L. & Block, S.M. Direct observation of cotranscriptional folding in an adenine riboswitch. Science 338, 397-400 (2012).
2. Portman, D.S. & Dreyfuss, G. RNA annealing activities in HeLa nuclei. EMBO J 13, 213-21 (1994).
3. Herschlag, D. RNA chaperones and the RNA folding problem. J Biol Chem 270, 20871-4 (1995).
4. Chu, C. et al. Systematic discovery of Xist RNA binding proteins. Cell 161, 404-16 (2015).
5. Cerase, A., Armaos, A., Cid-Samper, F., Avner, P. & Tartaglia, G.G. Xist lncRNA forms silencing granules that induce heterochromatin formation and repressive complexes recruitment by phase separation. bioRxiv, 351015 (2018).

Supplementary Methods

Supplementary Note 1 – For the analysis of fRIP-seq data

1. Download data from sra using the following standard format: /sra/sra-instant/reads/ByRun/sra/{SRR|ERR|DRR}/<first 6 characters of accession>/<accession>/<accession>.sra. For example: wget ftp://ftp-trace.ncbi.nih.gov/sra/sra-instant/reads/ByRun/sra/SRR/SRR197/SRR1976881/SRR1976881.sra

2. Convert sra to fastq using fastq-dump in the sra-toolkit, convert from paired end fastq to gapped fastq using the pe2gap.py script, and then combine the multiple fastq files for each sample (for example, concatenate SRR1976598-SRR1976603 to ADARrep1). The pe2gap.py script converts the second half of each 62nt read to its reverse complement.

```
for file in SRR*sra; do (fastq-dump $file &); done
for file in *fastq; do (python pe2gap.py 32 $file ${file%fastq}gap.fastq &); done
```

3. Map the combined fastq files to hg38 with the following specific parameters. The limitOutSJcollapsed option is adjusted to accommodate the large number of ‘splice junctions’ because essentially all the gapped reads are like ‘splice junctions’. The chimSegmentMin option allows chiastic mapping.

```
for file in *gap.fastq; do (star-static --runMode alignReads --genomeDir hg38/ --readFilesIn $file --outFileNamePrefix ${file%.fastq}_hg38 --outReadsUnmapped Fastq --outFilterMultimapNmax 1 --outSAMattributes All --alignIntronMin 1 --chimSegmentMin 15 --chimJunctionOverhangMin 15 --limitOutSJcollapsed 3000000 --runThreadN 8 &); done
```

4. Use samtools to convert, sort and index the star output sam files

```
for file in *gap_hg38Aligned.out.sam; do (samtools view -bS -o ${file%out.sam}bam $file; samtools sort ${file%out.sam}bam ${file%out.sam}_sorted; samtools index ${file%out.sam}_sorted.bam &); done
for file in *gap_hg38Chimeric.out.sam; do (samtools view -bS -o ${file%out.sam}bam $file; samtools sort ${file%out.sam}bam ${file%out.sam}_sorted; samtools index ${file%out.sam}_sorted.bam &); done
```

5. Convert the reads mapped to XIST in hg38 back to fastq. Note the output redirection is different for the two files.

```
for file in *gap_hg38Aligned_sorted.bam; do (samtools view $file chrX:73820651-73852753 | awk '{print "@" $1 "\n" $10 "\n+\n" $11}' > ${file%Aligned_sorted.bam}XIST.fastq &); done
for file in *gap_hg38Chimeric_sorted.bam; do (samtools view $file chrX:73820651-73852753 | awk '{print "@" $1 "\n" $10 "\n+\n" $11}' >> ${file%Chimeric_sorted.bam}XIST.fastq &); done
```

6. Map XIST reads to hsXIST ‘mini-genome’, which consists of the human mature XIST RNA sequence, with the following specific parameters. See previous publication on how the mini-genome was made [27].

```
for file in *gap_hg38XIST.fastq; do (star-static --runMode alignReads --genomeDir starhsXIST/ --readFilesIn $file --outFileNamePrefix ${file%hg38XIST.fastq}hsXIST --outReadsUnmapped Fastq --outFilterMultimapNmax 1 --
```

outSAMattributes All --alignIntronMin 1 --chimSegmentMin 15 --chimJunctionOverhangMin 15 --runThreadN 8 &); done

7. Use samPairingCalling.test.pl (from https://github.com/qczhang/) to convert the chiastic reads to normal gapped reads. This step is only used to combine the two files, not to assemble the duplex groups. The duplex group information is not used in the analysis of the five groups of long-distance reads.
for file in *gap_hsXISTAligned.out.sam; do (perl samPairingCalling.test.pl -i $file -j ${file%Aligned.out.sam}Chimeric.out.junction -s ${file%Aligned.out.sam}Chimeric.out.sam -o ${file%Aligned.out.sam}_geometric -g starhsXIST/hsXIST.fa -z starhsXIST/chrNameLength.txt -a annotations/empty.gtf -t starhsXIST/hsXIST.fa -l 15 -p 2 -c geometric 1>${Aligned.out.sam}_geometric.stdout 2>${Aligned.out.sam}_geometric.log &); done

8. Remove improperly assembled DGs (duplex groups), which are caused by lack of sufficient support reads.
for file in *gap_hsXIST_geometricsam; do (grep -v "DG:i:$" $file > ${file%sam}.sam &); done

9. Use readspan.py to extract the long pairs from the *gap_hsXIST_geometric.sam files and plot all the length distributions, output long.sam, shortdist.pdf and longdist.pdf. The cutoff is set at 1000nt to ensure only proximity-ligated fragments are extracted, given the length of average RNA fragments less than 200nt. The pdf files are assembled into multi-panel figures (Figure 1 and S1).
for file in *gap_hsXIST_geometric.sam; do (python readspan.py ${file%gap_hsXIST_geometric.sam} $file ${file%.sam}_long.sam ${file%.sam}_shortdist.pdf ${file%.sam}_longdist.pdf &); done

10. Make bedgraph files for visualization on IGV. The following is the script for automated processing of all samples. These tracks are combined with the arcs produced below (step 11) into multi-panel figures (Figure 1 and S1).
for file in *gap_hsXIST_geometric*.sam; do (samtools view -bS -o ${file%sam}bam $file; samtools sort ${file%sam}bam ${file%.sam}_sorted; samtools index ${file%.sam}_sorted.bam; genomeCoverageBed -ibam ${file%.sam}_sorted.bam -bg -split -g hsXIST.size > ${file%sam}bedgraph &); done

11. Use cigar2helixbed.py to convert the *gap_hsXIST_geometric_long.sam to bed files to visualize the arcs in the IGV genome browser. See IGV documents for the instructions on the visualization (https://software.broadinstitute.org/software/igv/node/284).
for file in *gap_hsXIST_geometric_long.sam; do (python cigar2helixbed.py $file ${file%.sam}_arc.bed &); done

12. Use the frip_subset_paris.py script to group the long-distance read pairs as follows. Given that only five major groups are discernable, reads are grouped by their anchor locations. The long-distance groups (LGs) are visualized together with the overlapping DGs from PARIS data (from step 13) in Figure 1.
for file in *gap_hsXIST_geometric_long.sam; do (python frip_subset_paris.py $file ${file%.sam}anchors.sam; samtools view -bS -o ${file%.sam}anchors.bam ${file%.sam}group.sam; samtools sort ${file%.sam}anchors.bam ${file%.sam}anchors_sorted; samtools index ${file%.sam}anchors_sorted.bam &); done

13. Use the frip_subset_paris.py script to extract PARIS DGs that are in the same region as the 5 fRIP-seq long-distance groups (LGs) as follows.

```
for file in AMT_Stress_trim_nodup_starhsXIST_l15p2_geometricNGmin.sam; do (python frip_subset_paris.py $file
${file%.sam}anchors.sam; samtools view -bS -o ${file%.sam}anchors.bam ${file%.sam}group.sam; samtools sort
${file%.sam}anchors.bam ${file%.sam}anchors_sorted; samtools index ${file%.sam}anchors_sorted.bam &); done
```

14. The 31nt sequencing tags do not represent the actual binding sites of the proteins; instead, the tags need to be extended to the size of the RNA fragments (~150nt) to reveal the approximate location of the protein binding and crosslinking. To make the extended fRIP-seq profiles for the long-distance pairs, the following script was used: frip_extend_longpairs.py. The data are visualized in Figure 1 and S1.

```
python frip_extend_longpairs.py 119 EZH2rep1gap_hsXIST_geometric_longanchors_LG2.sam
EZH2rep1gap_hsXIST_geometric_longanchors_LG2extend.sam
```

15. To normalize the bedgraph files for visualization on IGV, we made one copy of Input1rep1 control for each IP, since the level of enrichment is very different, a single normalization would not work. For example, for HNRNPU we use the first 800nt given the clustered binding in the large BCD and exon6 domains. The count for Input1rep1 is 664, and for HNRNPUrep1 is 66. Therefore the ratio is 0.099. For CBX3, we used the region 2000nt to 10000nt as follows and the normalization factor is 0.298

```
awk '($4>2000)&&($4<10000)' Input1rep1gap_hsXIST_geometric.sam | wc -l
awk '{print $1 "\t" $2 "\t" $3 "\t" $4*0.298}' CBX3rep1gap_hsXIST_geometric.bedgraph >
CBX3rep1gap_hsXIST_geometric_norm.bedgraph
```

16. To cluster the profiles of fRIP-seq experiments, the following commands are used. First we made 100nt windows and calculated coverage in 100nt intervals. Then all the files were combined to generate a matrix for all fRIP-seq profiles.

```
bedtools makewindows -g hsXIST.size  -w 100 > hsXIST_100nt.bed
bedtools coverage -split -abam ADARrep1gap_hsXIST_geometric_sorted.bam -b hsXIST_100nt.bed | sort -k2 -n >
ADARrep1gap_hsXIST_geometric_100nt.bed
for file in *gap_hsXIST_geometric_sorted.bam; do (bedtools coverage -split -abam $file -b hsXIST_100nt.bed | sort -
k2 -n | cut -f4 > ${file%sorted.bam}100nt.vector &); done
awk '{print $1 "_" $2 "_" $3}' ADARrep1gap_hsXIST_geometric_100nt.bed >
frip_gap_hsXIST_geometric_100nt.intervals
```

add a column name to the frip_gap_hsXIST_geometric_100nt.matrix file

```
for file in *gap_hsXIST_geometric_sorted.bam; do (echo ${file%gap_hsXIST_geometric_sorted.bam} | tr '\n' '\t' >>
frip_gap_hsXIST_geometric_100nt.matrix); done
```

add a new line to the end of the frip_gap_hsXIST_geometric_100nt.matrix file

```
paste frip_gap_hsXIST_geometric_100nt.intervals *vector >> frip_gap_hsXIST_geometric_100nt.matrix
```

17. To normalize the frip_gap_hsXIST_geometric_100nt.matrix file against input controls, we first divided the values of each bin in each sample by the values of the average of Input1rep1-Input1rep3. Then we adjusted the 25th percentile of each sample to 0.1. This step was performed using the script frip_norm.py.

18. After the matrix file was normalized, the clustering was performed using Cluster 3.0 and Java TreeView [61,62]. Cluster 3.0 options: Hirarchical, Arrays Cluster, correlation (uncentered), complete linkage. TreeView options as

follows. Settings: Pixel settings: Conrast 3.0. Positive black, zero white. Export to postscript, Gene Headers: NAME, Array Headers: Interval, Below tree: yes. Include: Array Tree, x scale 7, y scale 3.5, Border 0.

19. To plot the RIP enrichment of each sample in 100nt bins, replicates for each sample were averaged, sorted in ascending order and then plotted in Excel.

Supplementary Note 2 – For the analysis of eCLIP data

1. Download the bigWig files for all K562 eCLIP data from ENCODE (https://www.encodeproject.org/search/?type=Experimentl) using the following selection criteria: Assay: eCLIP, Experiment status: released, Biosample type: immortalized cell line, Life stage: adult, Available data: bigWig, and the following standard command: xargs -n 1 curl -O -L < files.txt. The downloaded bigWig files were renamed using the metadata.tsv file linked within the files.txt file.

For data that were mapped to hg19, they were converted to hg38 using eclip_rename.py as follows.
for file in *hg19.bw; do (bigWigToBedGraph $file ${file%bw}bedgraph &); done
for file in *hg19.bedgraph; do (liftOver $file ~/annotations/hg19ToHg38.over.chain ${file/_hg19/} ${file}unmapped &); done
for file in *neg.bedgraph *pos.bedgraph; do (sort -k1,1 -k2,2n $file > ${file%.bedgraph}sorted.bedgraph &); done
for file in *sorted.bedgraph; do (python ~/bin/liftover_clean.py $file ${file%.bedgraph}clean.bedgraph &); done
for file in *clean.bedgraph; do (/seq/ucsc/bedGraphToBigWig $file ~/annotations/hg38_chrom.sizes ${file%sortedclean.bedgraph}.bw &); done

2. Use the script eclip_bigwig2bedgraph.py to extract data for each RNA. This script produces one multibedgraph file with all data and all the extracted bedgraph files for each input bw file.
python ~/bin/eclip_bigwig2bedgraph.py XIST K562 . hsXIST.bed eCLIP_K562_XIST.multibedgraph &
python ~/bin/eclip_bigwig2bedgraph.py MALAT1 K562 . hsMALAT1.bed eCLIP_K562_MALAT1.multibedgraph &
python ~/bin/eclip_bigwig2bedgraph.py NEAT1 K562 . hsNEAT1.bed eCLIP_K562_NEAT1.multibedgraph &
python ~/bin/eclip_bigwig2bedgraph.py MALAT1 HepG2 . hsMALAT1.bed eCLIP_HepG2_MALAT1.multibedgraph &
python ~/bin/eclip_bigwig2bedgraph.py NEAT1 HepG2 . hsNEAT1.bed eCLIP_HepG2_NEAT1.multibedgraph &

3. Make a header file for all 121 K562 and 103 HepG2 RBP eCLIP experiments.
ls K562_*_XIST_?.bedgraph | sed 's/_XIST//g' | sed 's/\.bedgraph//g' | sed 's/_0/_SMInput/g' | sed 's/_1/_eCLIP1/g' | sed 's/_2/_eCLIP2/g' | tr '\n' '\t' > header_K562_363.txt
ls HepG2*MALAT1_?.bedgraph | sed 's/_MALAT1//g' | sed 's/\.bedgraph//g' | sed 's/_0/_SMInput/g' | sed 's/_1/_eCLIP1/g' | sed 's/_2/_eCLIP2/g' | tr '\n' '\t' > header_HepG2_309.txt

4. Then use the eclip_normalize.py to normalize against all controls. Given that the values in the bedgraph files are not the read numbers, I took the average of all the files as the background (121 for K562 and 103 for HepG2).
python eclip_normalize.py XIST 100 header_K562_363.txt eCLIP_K562_hsXIST_100nt.multibedgraph
Note, the bigWig files were made using the following scripts from Yeo lab, and essentially each file is normalized as number of reads per million. Then combine all the binned bedgraph files.

https://github.com/YeoLab/gscripts/blob/master/gscripts/general/make_bigwig_files.py
https://github.com/YeoLab/gscripts/blob/master/gscripts/general/normalize_bedGraph.py

The output files from steps 2-4 have the following dimensions:

Dimensions (rows x columns)	1nt.multibedgraph	100nt.multibedgraph
eCLIP_K562_hsXIST	19296x336	193x366
eCLIP_K562_hg38MALAT1	8706x336	88x366
eCLIP_K562_hg38NEAT1	22742x336	228x366
eCLIP_HepG2_hg38MALAT1	8706x312	88x312
eCLIP_HepG2_hg38NEAT1	22742x312	228x312

4. Note, editing large pdf or svg files in Illustrator is very slow. Here are some tricks to improve the performance. https://helpx.adobe.com/illustrator/kb/optimize-illustrator-performance-mac-os.html. Make a pdf file in Illustrator to store the names of the 121 samples for K562, and 103 samples for HepG2. This will replace the long file names for the tracks.

```
tr '\t' '\n' < header_K562_363.txt | grep eCLIP1 | sed 's/_eCLIP1//g'
tr '\t' '\n' < header_HepG2_309.txt | grep eCLIP1 | sed 's/_eCLIP1//g'
```

6. The normalized matrix (*normmatrix) files were clustered using the city-block distance and single-linkage, and visualized in TreeView, exported at x:2 y:1.

Reasons for choosing the parameters in clustering are as follows.

A. Although the vectors to be clustered are ratios, no log transformation is used because only the positive enrichment is meaningful.

B. Only arrays (here RBP eCLIP profiles) are normalized, because assessing the overall pattern similarity is high priority in clustering.

C. The arrays are not centered, again because only positive enrichment is meaningful

D. The correlation based similarity metrics are not appropriate because the magnitude of enrichment matters in this calculation.

E. The downside of the Euclidean or city-block distance is that similar patterns may be separated due to the difference in magnitude.

7. Then use the pc2track.py command to make principal component tracks for visualization. The top seven tracks were shown for the three lncRNAs

```
python pca2tracks.py eCLIP_K562_hsXIST_all121_100nt_pca_array.pc.txt 7 array
eCLIP_K562_hsXIST_all121_100nt_pca_array
python pca2tracks.py eCLIP_K562_hg38MALAT1_all121_100nt_pca_array.pc.txt 7 array
eCLIP_K562_hg38MALAT1_all121_100nt_pca_array
python pca2tracks.py eCLIP_K562_hg38NEAT1_all121_100nt_pca_array.pc.txt 7 array
eCLIP_K562_hg38BEAT1_all121_100nt_pca_array
```

8. Use this script to convert the PARIS data in bed format to chr1 based mature transcript: genome2transcript.py. This can be used to compare with the eCLIP data.

Supplementary Note 3 - Analysis of mouse NPC PIRCh data

1. The PIRCh paired end reads were first combined to form gapped reads as follows before mapping. The petwo2gap.py script makes the reverse complement of read 2 in each pair and append that to the end of read 1.
python petwo2gap.py NPC_H3K4Me3_rep1_R1.fastq NPC_H3K4Me3_rep1_R2.fastq NPC_H3K4Me3_rep1.fastq &

2. Map reads to mm10. Many of the mapped reads have insertions (I in the CIGAR string), suggesting that the two reads are overlapping on each fragment. The mapping statistics are included as follows for both NPC and human FL3 PIRCh data.
for file in NPC*.fastq.gz; do (star-static --readFilesCommand gunzip -c --runMode alignReads --genomeLoad LoadAndKeep --outFilterScoreMinOverLread 0.33 --outFilterMatchNminOverLread 0.33 --scoreGapNoncan 0 --scoreGapGCAG 0 --scoreGapATAC 0 --scoreInsOpen 0 --scoreInsBase 0 --alignSplicedMateMapLminOverLmate 0.33 --genomeDir starmm10 --readFilesIn npc_fastq/$file --outFileNamePrefix npc_fastq/${file%.fastq.gz}_mm10 --outReadsUnmapped Fastx --outFilterMultimapNmax 1 --outSAMattributes All --alignIntronMin 1 --chimSegmentMin 15 --chimJunctionOverhangMin 15 --limitOutSJcollapsed 3000000 --runThreadN 8 &); done

Samples	Total	Unique and (%)	Too many loci and (%)
NPC_H3K27Ac_rep1.fastq.gz	49789075	38554298 77.44%	7263690 14.59%
NPC_H3K27Ac_rep2.fastq.gz	74126049	56386602 76.07%	11958468 16.13%
NPC_H3K27Me3_rep1.fastq.gz	58730198	40141231 68.35%	9304105 15.84%
NPC_H3K27Me3_rep2.fastq.gz	50097333	38159419 76.17%	7995706 15.96%
NPC_H3K4Me3_rep1.fastq.gz	47341615	36277437 76.63%	7268422 15.35%
NPC_H3K4Me3_rep2.fastq.gz	69344567	53836981 77.64%	11111068 16.02%
NPC_IgG_rep1.fastq.gz	46986293	38158603 81.21%	6766164 14.40%
NPC_IgG_rep2.fastq.gz	59936890	47485939 79.23%	8639650 14.41%
NPC_Input_rep1.fastq.gz	50582511	41505853 82.06%	7820731 15.46%
NPC_Input_rep2.fastq.gz	62252561	51160069 82.18%	9779852 15.71%

3. Convert all files to bam.
for file in NPC*sam; do (samtools view -bS -o ${file%out.sam}bam $file &); done
for file in NPC*bam; do (samtools sort -o ${file%.bam}_sorted.bam $file &); done
for file in NPC*sorted.bam; do (samtools index $file &); done

4. Convert Xist mapped reads back to fastq. Note the redirection is different for the two files.
for file in *Aligned_sorted.bam; do (samtools view $file chrX:103460373-103483233 | awk '{print "@" $1 "\n" $10 "\n+\n" $11}' > ${file%Aligned_sorted.bam}Xist.fastq &); done
for file in *Chimeric_sorted.bam; do (samtools view $file chrX:103460373-103483233 | awk '{print "@" $1 "\n" $10 "\n+\n" $11}' >> ${file%Chimeric_sorted.bam}Xist.fastq &); done

5. Given that the combined paired end reads would have insertions (as defined by the SAM CIGAR tag "I") due to the partial overlap of the two reads, I edited the fastq files to make each read shorter, e.g. to 40nt, so that the insertions will be avoided. This processing is performed as follows: for file in *mm10Xist.fastq; do (cut -c1-40,113-152 $file > ${file/Xist/Xist80nt} &); done

6. Map Xist reads to mmXist with the following specific parameters. For mapping to the small genome, the scoring system was changed to increase penalty from 0 to -30 for gap opening --scoreGap. for file in NPC*Xist80nt.fastq.gz; do (star-static --readFilesCommand gunzip -c --runMode alignReads --genomeLoad LoadAndKeep --outFilterScoreMinOverLread 0.33 --outFilterMatchNminOverLread 0.33 --scoreGap -30 --scoreGapNoncan 0 --scoreGapGCAG 0 --scoreGapATAC 0 --scoreInsOpen 0 --scoreInsBase 0 --alignSplicedMateMapLminOverLmate 0.33 --genomeDir starmmXist --readFilesIn npc_fastq/$file --outFileNamePrefix npc_fastq/${file%10Xist80nt.fastq.gz}Xist --outReadsUnmapped Fastx --outFilterMultimapNmax 1 --outSAMattributes All --alignIntronMin 1 --chimSegmentMin 15 --chimJunctionOverhangMin 15 --limitOutSJcollapsed 3000000 --runThreadN 8 &); done

7. Use samPairingCalling.test.pl to convert the chiastic reads to normal gapped reads [27]. This step is only used to combine the two files, not to assemble the duplex groups. Instead given that only three major groups are discernable, reads are grouped by their span.
for file in $(ls npc_fastq/*mmXistAligned.out.sam | cut -d'/' -f7); do (perl samPairingCalling.test.pl -i npc_fastq/$file -j npc_fastq/${file%Aligned.out.sam}Chimeric.out.junction -s npc_fastq/${file%Aligned.out.sam}Chimeric.out.sam -o npc_fastq/${file%Aligned.out.sam}_geometric -g starmmXist/mmXist.fa -z starmmXist/chrNameLength.txt -a empty.gtf -t starmmXist/mmXist.fa -l 15 -p 2 -c geometric 1>npc_fastq/${file%Aligned.out.sam}_geometric.stdout 2>npc_fastq/${file%Aligned.out.sam}_geometric.log &); done

10. Make bedgraph for visualization on IGV. The bedgraph files can be batch loaded from Finder, using the search function. The following is the script for automated processing of all samples.
for file in *geometric.sam; do (samtools view -bS -o ${file%sam}bam $file; samtools sort ${file%sam}bam ${file%.sam}_sorted; samtools index ${file%.sam}_sorted.bam; genomeCoverageBed -ibam ${file%.sam}_sorted.bam -bg -split -g mmXist.size > ${file%sam}bedgraph &); done

11. To normalize the PIRCh data, use the script pirch_normalize.py. Input: individual bedgraph files directly derived from the bam data, and a header file of all the samples (pirch_NPC_header.txt, e.g. NPC_Input_rep1). Output: normalized in 100nt windows.
python pirch_normalize.py hg38XIST 100
for file in *norm100nt.bedgraph; do (sed 's/hg38/hs/g' $file > ${file/mm10/mm} &); done

12. Take the geometric mean for each pair of duplicates to remove the noise. For example
paste NPC_H3K27Ac_rep?_mmXist_geometric_norm100nt.bedgraph | awk '{print $1, '\t', $2, '\t', $3, '\t', ($4*$8)**0.5}' > avg_NPC_H3K27Ac_mmXist_geometric_norm100nt.bedgraph

13. Then the PIRCh data are lifted to the hsXIST coordinates to facilitate comparison with the human HNRNPU fRIP, eCLIP, and other related data:

for file in avg*; do (liftOver -minMatch=0.2 -minBlocks=0.2 -fudgeThick $file mmtohsXIST.liftoverchain ${file/.bedgraph/_hsXIST.bedgraph} unmapped &); done

Supplementary Note 4 - Analysis of human FL3 PIRCh data

1. The following three FL3 PIRCh data files were obtained in bigwig format mapped to hg19: FL3_H3_PA_nugen.bw, FL3_IgG_PA_nugen.bw and FL3_Input_nugen.bw. The data were lifted to hg38 using the liftOver utility from UCSC.

2. RNAs were extracted from the bw files using bigWigToBedGraph:
for file in FL3*hg38.bw; do (bigWigToBedGraph -chrom=chrX -start=73820650 -end=73852753 $file ${file%.bw}_XIST.bedgraph &); done

3. Normalization of the FL3 PIRCh data was performed using the same method described above (pirch_normalize.py).

Supplementary Note 5 - Analysis of mouse PARIS data

1. Sequencing was performed on a NextSeq together with PHIX spikein. For this PARIS library, PHIX reads were removed based on barcode information. Two sequencing runs were performed for the PARIS libraries, generating hatx1.fastq and hatx2.fastq. Then duplicates were removed: /home/zhipeng/bin/readCollapse -U hatx.fastq -O hatx_nodup.fastq &

2. Trim the 5' and 3' end adapter sequence:
java -jar trimmomatic-0.32.jar SE -threads 16 -phred33 hatx_nodup.fastq hatx_trim_nodup.fastq ILLUMINACLIP:P6SolexaRC35.fa:3:20:10 HEADCROP:16 MINLEN:20 &

3. Quality of the processed files was visualized using fastqc.

4. Map reads to the mm10 reference. Given that mouse Xist contains multiple nearly identical repeats, I allowed multiple mapping in this run (--outFilterMultimapNmax 20).
star-static --runMode alignReads --runThreadN 16 --genomeDir starmm10 --genomeLoad LoadAndKeep --readFilesIn hatx_trim_nodup.fastq --limitOutSJcollapsed 3000000 --outFileNamePrefix hatx_trim_nodup_mm10 --outReadsUnmapped Fastx --outSAMattributes All --outFilterMultimapNmax 20 --outFilterScoreMinOverLread 0.33 --outFilterMatchNminOverLread 0.33 --scoreGapNoncan 0 --scoreGapGCAG 0 --scoreGapATAC 0 --scoreInsOpen 0 --scoreInsBase 0 --alignIntronMin 1 --alignSplicedMateMapLminOverLmate 0.33 --chimSegmentMin 15 --chimJunctionOverhangMin 15

5. Convert Xist mapped reads back to fastq. Note the redirection is different for the two files. Since that some of the reads are output to both the Aligned.out.sam and Chimeric.out.sam files, we need to take the unique reads for mapping to mmXist.
samtools view hatx1_trim_nodup_mm10Aligned_N_sorted.bam chrX:103460373-103483233 | awk '{print "@" $1 "\n"

```
$10 "\n+\n" $11}' > hatx_mm10Xist1.fastq
samtools view hatx1_trim_nodup_mm10Chimeric_sorted.bam chrX:103460373-103483233 | awk '{print "@" $1 "\n" $10 "\n+\n" $11}' > hatx_mm10Xist2.fastq
samtools view hatx2_trim_nodup_mm10Aligned_sorted.bam chrX:103460373-103483233 | awk '{print "@" $1 "\n" $10 "\n+\n" $11}' > hatx_mm10Xist3.fastq
samtools view hatx2_trim_nodup_mm10Chimeric_sorted.bam chrX:103460373-103483233 | awk '{print "@" $1 "\n" $10 "\n+\n" $11}' > hatx_mm10Xist4.fastq
cat hatx_mm10Xist* > hatx_mm10Xist.fastq
awk 'NR%4 {printf "%s ",$0;next}1' hatx_mm10Xist.fastq > a; sort a | uniq -u > b
awk '{print $1 "\n" $2 "\n" $3 "\n" $4}' b > hatx_mm10Xist_uniq.fastq
```

6. Map Xist reads to mmXist with the following specific parameters. For mapping to the small mmXist “genome”, the --scoreGap option was changed from 0 to -30.

```
star-static --runMode alignReads --genomeLoad LoadAndKeep --outFilterScoreMinOverLread 0.33 --outFilterMatchNminOverLread 0.33 --scoreGap -10 --scoreGapNoncan 0 --scoreGapGCAG 0 --scoreGapATAC 0 --scoreInsOpen 0 --scoreInsBase 0 --alignSplicedMateMapLminOverLmate 0.33 --genomeDir starmmXist --readFilesIn hatx_mm10Xist_uniq.fastq --outFileNamePrefix hatx_mmXist_uniqp10 --outReadsUnmapped Fastx --outFilterMultimapNmax 1 --outSAMattributes All --alignIntronMin 1  --chimSegmentMin 15 --chimJunctionOverhangMin 15 --limitOutSJcollapsed 3000000 --runThreadN 8
```

7. Use samPairingCalling.test.pl to convert the chiastic reads to normal gapped reads.

```
perl samPairingCalling.test.pl -i hatx_mmXist_uniqp10Aligned.out.sam -j hatx_mmXist_uniqp10Chimeric.out.junction -s hatx_mmXist_uniqp10Chimeric.out.sam -o hatx_mmXist_uniqp10geometric -g starmmXist/mmXist.fa -z /home/zhipeng/starmmXist/mmXist.size -a empty.gtf -t starmmXist/mmXist.fa -l 15 -p 2 -c geometric 2>hatx_mmXist_uniqp10geometric_log.txt
```

8. Convert the output DG information to bed format for visualization in IGV.

```
samtools view -bS -o hatx_mmXist_uniqp10geometricbam hatx_mmXist_uniqp10geometricsam
samtools sort hatx_mmXist_uniqp10geometricbam hatx_mmXist_uniqp10geometric_sorted
samtools index hatx_mmXist_uniqp10geometric_sorted.bam
python dg2bed.py hatx_mmXist_uniqp10geometric hatx_mmXist_uniqp10geometric.bed bed12
```

9. To compare with the human XIST structure, I lifted the mouse coordinates to the human one hsXist. Of the 203 DGs (including ones with only identical reads), 108 can be lifted to human XIST coordinates using the adjusted liftOver parameters shown as follows based on previous tests [27].

```
liftOver -minMatch=0.2 -minBlocks=0.2 -fudgeThick hatx_mmXist_uniqp10geometric.bed mmtohsXIST.liftoverchain hatx_mmXist_uniqp10geometric_hsXIST.bed unmapped
```

10. To compare the mouse lifted PARIS data with the PARIS data from HEK293 cells, use the following scripts. This shuffling test (1000 times) showed that P<0.001.

```
cp AMT_Stress_trim_nodup_starhsXIST_l15p2_NGmin1386_arc.bed a12.bed
cp hatx_mmXist_uniqp10geometric_hsXIST.bed b12.bed
```

```
comparehelix.sh a12.bed b12.bed
dgshuffle.sh hsXISTsimp.bed /hsXIST.size commonlist
```

11. DG66 and DG69 in the mouse Xist PARIS data are consistent with the human XIST PARIS data [27], one of the most conserved long-range duplex. These two DGs were extracted as follows.

```
awk '($21~/DG:i:66/)||($21~/DG:i:69/)' hatx_mmXist_uniqp10geometricsam | cut -f4,6,10 > hatx_DG66DG69
```

Supplementary Note 6 - Global analysis of meRIP-seq data

The following input and immunoprecipitation paired-end (75nt x2 or 76nt x2) datasets were generated using the method described above. Five alleles were analyzed using MiSeq: wt, dsx (ΔSX, or A-repeat deletion), ki5, ki14 and ki17_1. All 6 alleles were analyzed by NextSeq: wt, dsx (ΔSX, or A-repeat deletion), ki5, ki14 and ki17_1 and ki17_2. The first 5 of the NextSeq libraries were re-sequencing of the same libraries for MiSeq. The general pipeline is as follows: map paired-end reads to mm10 → convert reads to bam format and separate the two strands → convert to bedgraph and bigwig for visualization. For global analysis: use m6Aviewer [63]. For specific analysis of Xist: extract data in the mouse Xist region → normalize against background ranges → normalize against wildtype input in 200nt windows → count coverage in each predefined m6A domains → plot m6A modification levels in bar graphs → compare known m6A motifs with actual modification sites as determined by meRIP-seq. Detailed analysis pipeline is described as follows.

Convert paired end reads to gap reads → map to hg38 → extract reads mapped to XIST → map to hsXIST 'minigenome' → assemble mapped Aligned and Chimeric reads → extract long pairs → make distance distribution, bedgraph and arcs for both short and long pairs.

1. Map paired-end reads to mm10 using the STAR (here *1.fastq indicate the first mate of each pair of paired-end files). for file in *1.fastq; do (star-static --runMode alignReads --genomeLoad LoadAndKeep --genomeDir starmm10/ --readFilesIn $file ${file/1.fastq/2.fastq} --outFileNamePrefix ${file/1.fastq/_mm10} --outReadsUnmapped Fastx --outFilterMultimapNmax 1 --outSAMattributes All --alignIntronMin 20 --runThreadN 6 &); done

2. Convert to bam files and separate the strands

```
for file in *sam; do (samtools view -bS -o ${file/out.sam/bam} $file &); done
for file in *bam; do (samtools sort -o ${file/.bam/_sorted.bam} $file &); done
for file in *sorted.bam; do (samtools index $file &); done

for file in *_mm10Aligned_sorted.bam; do (
samtools view -b -f 128 -F 16 $file > ${file}fwd1.bam; samtools view -b -f 80 $file > ${file}fwd2.bam;
samtools view -b -f 144 $file > ${file}rev1.bam; samtools view -b -f 64 -F 16 $file > ${file}rev2.bam;
samtools merge ${file/sorted.bam/pos.bam} ${file}fwd1.bam ${file}fwd2.bam;
samtools merge ${file/sorted.bam/neg.bam} ${file}rev1.bam ${file}rev2.bam;
samtools index ${file/sorted.bam/pos.bam}; samtools index ${file/sorted.bam/neg.bam}); done &
```

3. Convert bam to bedgraph and then bw for visualization. for file in *pos.bam *neg.bam; do (bedtools genomecov -bg -split -ibam $file -g starmm10/chrNameLength.txt > ${file/bam/bedgraph} &); done
for file in *bedgraph; do (bedGraphToBigWig $file starmm10/chrNameLength.txt ${file/bedgraph/bw} &); done

4. Use the m6aViewer software (version 1.6.1) to calculate global changes in m6A modification using the default parameters and negative strand bam files (half the data). To retrieve the columns that contain the dsx or wildtype comparisons, for example, use the following script: head -n 1 All6_differential.txt | awk 'BEGIN {FS="\t"}; { for (i=1; i<=NF; ++i) { if ($i ~ "dsx") print i } }'

5. Plot the differences using scatter_volcano.py script. Log scale fold changes (LFC) and log scale p values were used as the x-axis and y-axis, respectively. For example: python scatter_volcano.py All6_differential.txt 29 30 m6A_global_scatter_dsx_vs_ki14.png

Targeted analysis of Xist m6A modification

1. Continuing from step 3 of the global analysis of meRIP-seq, extract data from the mm10 reference to mmXist coordinates using eclip_bigwig2bedgraph.py and normalize data against input using pirch_normalize.py. python ~/bin/eclip_bigwig2bedgraph.py mm10Xist bw . 1 mmXist.bed TXYmerip_mmXist.multibedgraph

2. Convert the “chr1” to “mmXist”: awk '{print "mmXist\t" $2 "\t" $3 "\t" $4}' XXX_neg_mm10Xist_1.bedgraph > XXX_neg_mmXist_1.bedgraph

3. For normalization, use the following ranges as background: 2000-4500, 5500-9000, 13000-14000 and 16000-17000. The files are normalized so that the wildtype input is set as the baseline. for file in *all*mm10Xist*bedgraph; do (echo $file; awk '($2>=2000)&&($2<4500)||($2>=5500)&&($2<9000)||($2>=13000)&&($2<14000)||($2>=16000)&&($2<17000) {sum+=$4}; END {print sum}' $file); done

4. Normalize each file so that coverage in the background ranges are the same as the wildtype input: awk '{print "mmXist\t" $2 "\t" $3 "\t" $4*norm_factor}' XXX_inall_mm10Aligned_neg_mm10Xist_1.bedgraph > XXX_inall_mm10Aligned_neg_mmXist_normbgrd.bedgraph

5. To visualize the bedgraph files in IGV, means were taken in 50 nt windows to minimize file size.

6. Count the reads in each predefined domain for making the bar graph of m6A levels. The domains were defined as follows: m6AD1: 0-1400, m6AKI5: 4500-5500, m6AD2: 9100-9900, m6AD3:11400-12300, m6AKI14:14100-15300, m6AD4/m6AKI17: 17000-17900. for file in *mmXist_normbgrd.bedgraph; do (bedtools map -c 4 -o sum -null 0 -a mmXist_m6AD.bed -b $file); done > mmXist_m6AD.count

7. m6A levels were normalized against wildtype and then plotted in MS Excel.

8. Used the script motif.py to make a track for all the m6A motifs in mmXist and a track of motif density in 300nt windows and 50nt steps. There are 333 DRACH motifs in total in the 17918nt mouse Xist transcript and 360 motifs in the 19296nt human XIST.
python motif.py DRACH mmXist.fa mmXist_DRACH.bed 300 50 mmXist_DRACH_density.bedgraph

Analysis of the silencing functions of Xist alleles using the meRIP-seq input data.

1. Summarize expression levels of all mouse genes. for file in *inall_mm10Aligned*.bam; do (bedtools coverage -s -split -abam $file -b ~/annotations/mm10refGenePU.bed > ${file/bam/count} &); done

2. Extract strand-specific count for all the input files and then combine them:
for file in *all*pos.count; do (awk '$6=="+"' $file > ${file}pos); done
for file in *all*neg.count; do (awk '$6=="+"' $file > ${file}neg); done
file in *all*countpos; do (cat $file ${file/pos.countpos/neg.countneg} > ${file%_pos.countpos}.countstrand); done

3. Summarize expression by chromosome. Combine the counts from all samples. paste *inall_mm10Aligned.countstrand | cut -f1-4,13,29,45,61,77,93 > Inputall_mm10Aligned.count

3. Take the ratios for each gene against wt, for genes with counts >=100. awk '($5>=100)&&($6>=100)&&($7>=100)&&($8>=100)&&($9>=100)&&($10>=100) {print $1 "\t" $2 "\t" $3 "\t" $4 "\t" $5/$10 "\t" $6/$10 "\t" $7/$10 "\t" $8/$10 "\t" $9/$10}' Inputall_mm10Aligned.count > Inputall_mm10Aligned_xci_violin.ratios

4. Plot the expression difference among all the samples using this script: violin_xci.py. The medians of ratios of expression levels all autosomal genes between mutant and wildtype were set to one for each mutant cell line, and then the medians of ratios for X chromosome genes were calculated. Differences between autosome and X chromosome ratios were assessed using Mann-Whitney U test.

Supplementary Discussion

The discovery of this modular topology raises many questions about the formation and function of XIST structures. While we have found a critical role of the XIST structure in determining RBP specificity, it is unclear how the long-range structures form in the first place. RNA structure formation is primarily driven by the stacking of base pairs. As RNA is transcribed, local structures can quickly form low energy conformations, and the co-transcriptional folding process is integral to the function of riboswitches in bacteria 1.

Thus mechanisms must exist to counter the tendency of local structure formation to allow long-range structures in XIST to form. Given the high abundance of many hnRNP proteins in the nucleus and their interactions with XIST (e.g. HNRNPK and HNRNPU), it is likely that the co-transcriptional binding of hnRNPs would compete with base pairing and contribute to the formation of observed high level architecture. Certain hnRNP proteins possess RNA annealing activities, and as a result may affect the observed structures by remodeling of co-transcriptionally formed duplexes 2,3. Numerous RNA helicases associate with the XIST RNA and likely play a role in remodeling XIST structure 4.

Multivalent interactions underlie phase separation, a general mechanism of organization in cells. Recently it was reported that the XIST RNP complex forms a distinct liquid phase in cells, probably due to the repetitive sequences in XIST and the intrinsically disordered regions in XIST-associated proteins 5. Our analysis showed that XIST associated proteins bind XIST in clustered patterns, supporting the multivalent interactions. Therefore, the phase separation process might contribute to the high affinity and specificity of XIST RNP formation.
