# Supplementary material for: Structural modularity of the XIST ribonucleoprotein complex
Source: Nat Commun. 2020 Dec 2;11:6163. doi: 10.1038/s41467-020-20040-3 (PMC7710737; doi:10.1038/s41467-020-20040-3)
Supplement: Supplementary file 3 — Reporting Summary [file 41467_2020_20040_MOESM3_ESM.pdf]

nature research

Corresponding author(s): Zhipeng Lu, Howard Y Chang

Last updated by author(s): Apr 25, 2020

Reporting Summary

Nature Research wishes to improve the reproducibility of the work that we publish. This form provides structure for consistency and transparency in reporting. For further information on Nature Research policies, see Authors & Referees and the Editorial Policy Checklist.

Statistics

For all statistical analyses, confirm that the following items are present in the figure legend, table legend, main text, or Methods section.

n/a	Confirmed	
☐	☒	The exact sample size (*n*) for each experimental group/condition, given as a discrete number and unit of measurement
☐	☒	A statement on whether measurements were taken from distinct samples or whether the same sample was measured repeatedly
☐	☒	The statistical test(s) used AND whether they are one- or two-sided<br>*Only common tests should be described solely by name; describe more complex techniques in the Methods section.*
☒	☐	A description of all covariates tested
☒	☐	A description of any assumptions or corrections, such as tests of normality and adjustment for multiple comparisons
☐	☒	A full description of the statistical parameters including central tendency (e.g. means) or other basic estimates (e.g. regression coefficient) AND variation (e.g. standard deviation) or associated estimates of uncertainty (e.g. confidence intervals)
☐	☒	For null hypothesis testing, the test statistic (e.g. *F*, *t*, *r*) with confidence intervals, effect sizes, degrees of freedom and *P* value noted<br>*Give P values as exact values whenever suitable.*
☒	☐	For Bayesian analysis, information on the choice of priors and Markov chain Monte Carlo settings
☒	☐	For hierarchical and complex designs, identification of the appropriate level for tests and full reporting of outcomes
☒	☐	Estimates of effect sizes (e.g. Cohen's *d*, Pearson's *r*), indicating how they were calculated

Our web collection on statistics for biologists contains articles on many of the points above.

Software and code

Policy information about availability of computer code

Data collection	No software was used for data collection
Data analysis	STAR 2.7.1a (Dobin et al., 2013) https://github.com/alexdobin/STAR. Samtools v1.1 (Li et al., 2009) http://samtools.sourceforge.net/. Bedtools v2.22.0 (Quinlan and Hall, 2010) https://bedtools.readthedocs.io/. m6aViewer v1.6.1 (Antanaviciute et al., 2017) http://dna2.leeds.ac.uk/m6a/. PARIS v1.0 (Lu et al., 2016) https://github.com/qczhang. IGV 2.7.0 (Robinson et al., 2011), http://broadinstitute.org/software/igv. Vienna RNA Package 2.0 (Lorenz et al., 2011) https://www.tbi.univie.ac.at/RNA/. Kent Utilities (Fujita et al., 2011) https://genome.ucsc.edu/util.html. Trimmomatic v0.3.2 (Bolger et al., 2014) http://www.usadellab.org/cms/?page=trimmomatic. Python 2.7 (Van Rossum, 1995) https://www.python.org/. Cluster 3.0 (de Hoon et al., 2004) http://bonsai.hgc.jp/~mdehoon/software/cluster/software.htm. Java Treeview v1.1 (Saldanha, 2004) http://jtreeview.sourceforge.net/. Fastqc 0.11.8 (Andrews, 2010) https://www.bioinformatics.babraham.ac.uk/projects/fastqc/. Cutadapt 2.0 (Martin et al. 2011). Bowtie2, version 2.3.2 (Langmead and Salzberg 2012). Custom scripts, https://github.com/zhipenglu.

For manuscripts utilizing custom algorithms or software that are central to the research but not yet described in published literature, software must be made available to editors/reviewers. We strongly encourage code deposition in a community repository (e.g. GitHub). See the Nature Research guidelines for submitting code & software for further information.

Data

Policy information about availability of data

All manuscripts must include a data availability statement. This statement should provide the following information, where applicable:

- Accession codes, unique identifiers, or web links for publicly available datasets
- A list of figures that have associated raw data
- A description of any restrictions on data availability

Deposited Data: fRIP-seq (Hendrickson et al., 2016) GEO: GSE67963. eCLIP (Van Nostrand et al., 2016) https://www.encodeproject.org/. LBR CLIP (Chen et al., 2016) GEO: GSE86250. m6A iCLIP (Linder et al., 2015) GEO: GSE63753. Human HEK293 cell PARIS data (Lu et al., 2016) GEO: GSE74353. Human and mouse PIRCh (Fang et

al., 2019) GEO: GSE119006. Conservation plot is imported from "100 vertebrates Basewise Conservation by PhyloP" at UCSC genome browser. Normalized bedgraph files for all 121 proteins in eCLIP and 25 proteins in fRIP-seq are available in https://www.dropbox.com/sh/24kbqwxafhhzrli/AAADwDA6gdDOY-hWFoS4-V3aa?dl=0. The custom IGV genome for human XIST mature transcript is available in the same folder as well. All raw sequencing reads and raw count matrices generated in this study are available through Gene Expression Omnibus (GEO) with accession number GSE126715 (m6A RIP-seq and irCLIP on A-repeat relocation alleles), GSE126716 (PARIS in mouse ES cells). Every figure has associated raw data and they have been provided in the Source Data files. There are no restrictions on the data availability.

Field-specific reporting

Please select the one below that is the best fit for your research. If you are not sure, read the appropriate sections before making your selection.

☒ Life sciences ☐ Behavioural & social sciences ☐ Ecological, evolutionary & environmental sciences

For a reference copy of the document with all sections, see nature.com/documents/nr-reporting-summary-flat.pdf

Life sciences study design

All studies must disclose on these points even when the disclosure is negative.

Sample size	No sample size calculation was performed. Sample sizes was determined to be adequate based on the magnitude and consistency of measurable differences between groups. N=3 was used for each sample since this was enough for the study.
Data exclusions	No data were excluded.
Replication	All replication attempts were successful, all by the same experimenter. Sequencing experiments were not repeated beyond what were reported. q-PCR experiments were repeated at least twice. The edited cell lines were only generated once, and one or two clones were used for each genotype.
Randomization	This is not relevant to our study, since all variables were explicitly declared and controlled. The results are not affected by allocation of samples.
Blinding	Blinding was not applicable to this study because the differences were obvious to the observer and the data were not subjective.

Reporting for specific materials, systems and methods

We require information from authors about some types of materials, experimental systems and methods used in many studies. Here, indicate whether each material, system or method listed is relevant to your study. If you are not sure if a list item applies to your research, read the appropriate section before selecting a response.

Materials & experimental systems

n/a	Involved in the study	
☐	☒	Antibodies
☐	☒	Eukaryotic cell lines
☒	☐	Palaeontology
☒	☐	Animals and other organisms
☒	☐	Human research participants
☒	☐	Clinical data

Methods

n/a	Involved in the study	
☒	☐	ChIP-seq
☒	☐	Flow cytometry
☒	☐	MRI-based neuroimaging

Antibodies

Antibodies used	Antibodies were not diluted, since they are used in immunoprecipitation experiments. Lot numbers were no longer available since most of them were used up.<br>Anti-N6-methyladenosine (m6A) antibody, Millipore Sigma, ABE572, https://www.emdmillipore.com/US/en/product/Anti-N6-methyladenosine-m6A-Antibody,MM_NF-ABE572.<br>SPEN antibody, Bethyl Laboratories, A301-119A, https://www.bethyl.com/product/A301-119A/SHARP+Antibody.<br>SPEN antibody, Abcam, Ab72266. https://www.abcam.com/spen-antibody-ab72266.html<br>LBR antibody, Protein Tech, 12398-1-AP. https://www.ptglab.com/Products/LBR-Antibody-12398-1-AP.htm<br>LBR antibody, Guttman laboratory (Chen et al. 2016 Science).
Validation	See validation on vendors websites listed above. For the LBR antibody from the Guttman lab, see Chen et al. 2016 Science. the m6A antibody was validated based on the enrichment of the canonical DRACH motif and RNAs that contain the modifications. The SPEN antibodies were validated based on specific enrichment of the A-repeat domain in XIST, according to previous studies.

Eukaryotic cell lines

Policy information about cell lines

Cell line source(s)	Experimental Models: HATX mES cell line Anton Wutz (Monfort et al., 2015). TXY mES cell line Edith Heard (Wutz et al., 2002) TXY:ΔSX mES cell line Edith Heard (Wutz et al., 2002). TXY:KI5, TXY:KI14, TXY:KI17_1, TXY:KI17_2 mES lines: this paper . K562 cells and HEK293T cells were from ATCC (CCL-243 and CRL3216).
Authentication	The cell lines contain Xist mutations and were confirmed by PCR, Sanger sequencing and high throughput sequencing.
Mycoplasma contamination	All cell lines tested negative for mycoplasma contamination
Commonly misidentified lines (See ICLAC register)	No commonly misidentified cell lines were used in this study
