# Supplementary material for: Structural modularity of the XIST ribonucleoprotein complex
Source: Nat Commun. 2020 Dec 2;11:6163. doi: 10.1038/s41467-020-20040-3 (PMC7710737; doi:10.1038/s41467-020-20040-3)
Supplement: Supplementary file 4 — Description of Additional Supplementary Files [file 41467_2020_20040_MOESM4_ESM.pdf]

Description of Additional Supplementary Files

File Name: Supplementary Data 1
Description: Enrichment of RBPs on XIST, and comparison to ChIRP-MS. The 121 RBPs studied by eCLIP were analyzed using window-based normalization against size-matched controls (See Supplementary Methods for details). The entire 19278nt human mature XIST is divided into 193 windows, each 100nt, and the enrichment values were ranked from low to high for each RBP. The geometric mean of enrichment ratios for the two biological replicates for each RBP were used for the ranking. The 25th percentile of non-zero enrichment values in each RBP was set to 0.1. Then the highest enrichment bin was used to rank all RBPs. Out of the 81 proteins previously detected by Xist ChIRP in mouse cells [22], 27 of them are included in the 121 eCLIP dataset, and highlighted. One of the RBPs, SRSF1, was reported by Royce-Tolland and colleagues [41]. There are 193 intervals, each representing a 100nt region along the human mature XIST RNA, for which enrichment was calculated.
